# Phytochemistry and Allelopathic Properties of Invasive *Heracleum sosnowskyi* Aqueous Extracts Against Lettuce (*Lactuca sativa* L.), Perennial Ryegrass (*Lolium perenne* L.), Timothy (*Phleum pratense* L.) and White Clover (*Trifolium repens* L.)

**DOI:** 10.3390/plants15030346

**Published:** 2026-01-23

**Authors:** Asta Judžentienė, Aistė Kundrotaitė, Tatjana Charkova, Irena Nedveckytė

**Affiliations:** 1 Institute of Biosciences, Life Sciences Center, Vilnius University, Saulėtekio Avenue 7, LT-10257 Vilnius, Lithuania; aiste.kundrotaite@gmc.stud.vu.lt (A.K.); irena.nedveckyte@gf.vu.lt (I.N.); 2 Department of Organic Chemistry, Center for Physical Sciences and Technology, Saulėtekio Avenue 3, LT-10257 Vilnius, Lithuania; tatjana.charkova@ftmc.lt

**Keywords:** *Heracleum sosnowskyi* Manden. (f. *Apiaceae*), extracts, GC/MS, HPLC/DAD/TOF, furanocoumarins, polyphenolic acids, flavonoids, allelopathy, inhibition, germination rate

## Abstract

*Heracleum sosnowskyi* is considered to be a dangerous invasive plant species that has successfully naturalized within a variety of plant communities across numerous countries. As a result of its superior competitiveness, the alien species is able to displace the indigenous species from their native habitats, thus changing the ecosystems and decreasing biodiversity. The phytochemicals present in the *H. sosnowskyi* aqueous extracts were revealed using GC/MS and HPLC/DAD/TOF techniques. Isopsoralen, methoxsalen, (iso)pimpinellin and/or bergapten were determined to be major compounds in the leaf, inflorescence and root extracts. Glutaric, quinic, linolenic, (iso)chlorogenic and other polyphenolic acids were identified in the extracts. Furthermore, a number of furanocoumarins, including hermandiol, bakuchicin, candinols (A and C) and candibirin F, and coumarins, umbelliferone and yunngnins (A and B), were identified in the roots. Additionally, the presence of flavonoids, including astragalin, quercetin 7,3,4-trimethyl ether, nicotiflorin and rutin, has been detected in the flower and leaf extracts. Allelopathic effects of *H. sosnowskyi* aqueous extracts were tested on four model plants, lettuce (*Lactuca sativa* L.) and three native Lithuanian meadow herbs, perennial ryegrass (*Lolium perenne* L.), timothy (*Phleum pratense* L.) and white clover (*Trifolium repens* L.), using the Petri dish method. *H. sosnowskyi* flower and leaf extracts demonstrated the strongest inhibitory effects on the germination and growth of the tested plant seeds. At the highest relative concentrations, 0.5 and 1.0, extracts of Sosnowsky’s hogweed inflorescences inhibited timothy seedling growth by 95.47% (from 19.64 ± 2.57 mm to 0.89 ± 0.73 mm) and 100%, respectively. The leaf extracts exhibited the strongest inhibitory effects on white clover seedlings. The highest relative concentrations tested (0.5 and 1.0) suppressed clover seedling growth by 94.66% (from 41.22 ± 2.53 mm to 2.20 ± 0.63 mm) and 100%, respectively. Additionally, the germination rate and vigor index of model plants were assessed. The research is of significance for the regulation and monitoring of the spreading of aggressive *H. sosnowskyi* plants. Moreover, it is important for the development of natural herbicides based on active phytotoxic compounds from these plants.

## 1. Introduction

**Figure 1:**
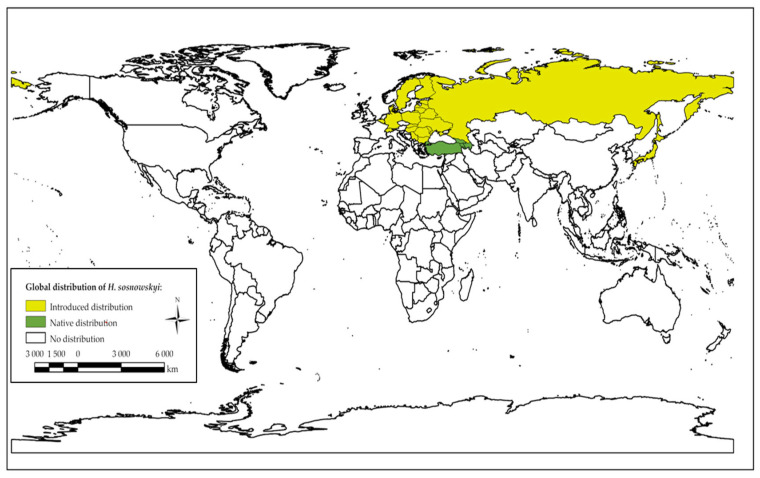
A visual representation of the global distribution of *Heracleum sosnowskyi* plants (up to 2024). The map was created using data from Plants of the World Online (POWO), World Flora Online (WFO) and the Global Biodiversity Information Facility (GBIF) [1,2,7] and ArcGIS Pro software (Version 3.5) (Esri, HNIT-BALTIC, Vilnius, Lithuania).

*Heracleum* (the family *Apiaceae*) is a widespread and taxonomically complex genus, containing numerous species distributed across the temperate regions of Asia, Africa, Europe and North America, with the highest centers of species diversity in China and the Caucasus [1,2,3]. The species name *Heracleum sosnowskyi* Manden. (also known as Sosnovskyi hogweed) was first published in 1944, with the species having been discovered in 1772 [4]. It is a monocarpic biennial or perennial herbaceous flowering plant species, native to the Caucasus (North Caucasus and Transcaucasia) and Western Asia (Turkey); and, in these regions, it has been observed to grow in the upper forest belt of the southern slopes, primarily in clearings, forest edges and meadows [1,2,5,6]. The *Heracleum* species has been introduced into Asia’s temperate climate regions (Russian Far East; Sakhalin and Kamchatka; Irkutsk, Siberia; West Siberia) and Europe (Eastern, Southeastern and Middle Europe; Central, East and North European Russia; Ukraine) [1,2,7] (Figure 1).

Sosnovskyi hogweed was first introduced from its native habitats to Russia in 1947, where it was cultivated as a highly productive fodder crop for livestock. Later, *H. sosnowskyi* spread to other countries, and it is now widely distributed on abandoned, non-cultivated agricultural lands, roadsides, gardening plots and forested areas in Germany, Hungary, Poland, Estonia, Latvia, Lithuania, Belarus, Ukraine and Russia [6,8,9,10,11].

**Figure 2:**
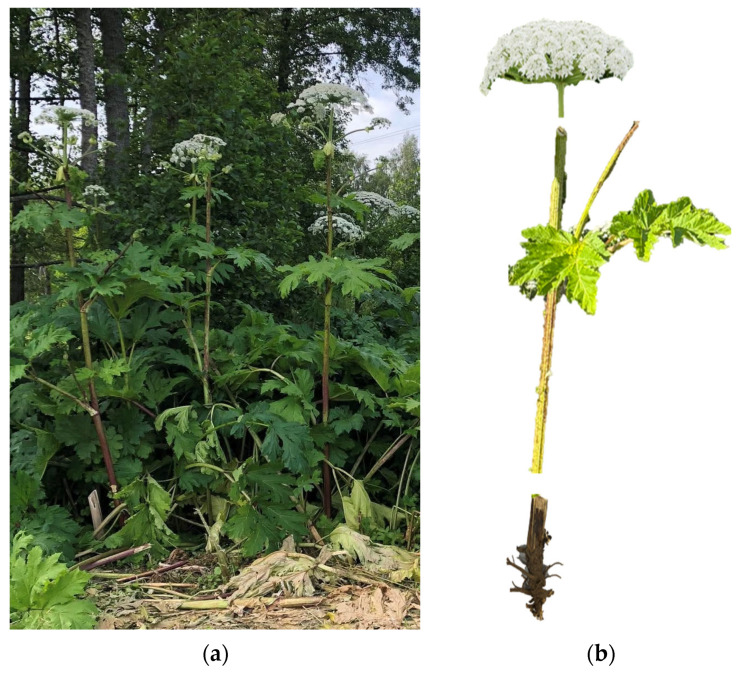
Imagines of *Heracleum sosnowskyi* plants (**a**) and its main parts (**b**) (by A. Kundrotaitė).

*H. sosnowskyi* exhibits several distinctive morphological characteristics (Figure 2). Among these is its considerable size: attaining heights of up to 3–5 m, it is among the tallest and largest herbaceous plants in Europe [5].

The erect, ridged and sparsely hairy stems with purple blotches of Sosnowskyi hogweed are very similar to those of giant hogweed (*Heracleum mantegazzianum*) [6]. The hollow stem has a diameter of 12 to 15 cm in the basal part, and it regrows from the large fleshy tap root in spring. The leaves of the mature plant are pinnately divided, with a length up to 1 m. Insect-pollinated, hermaphrodite flowers are arranged in compound umbels that mature in sequence. The lateral inflorescence diameter ranges between 25 and 32 cm [11,12] (Figure 2). Blossoming of white or pinkish inflorescences occurs in the second year or later (usually from July to September) [6]. Moreover, it is observed that the plant can survive for a period of up to six years before flowering, after which the mother plant dies once it has produced seeds. On average, a single plant is capable of producing approximately 9000 fruits. The total plant generative production was found to range between 7722 and 8082 mericarps, containing 15,444–16,164 seeds, across all habitats (both natural and anthropogenic), when assessed by Baležentienė et al. in Central Lithuania [13]. However, as posited by Moravcová et al., the viability of seeds is limited to a single season, primarily due to their quick germination in spring (95.2%) and the subsequent rapid decay of dormant seeds [14]. The root is strong and vigorous (up to 30 cm diameter) and can remain in the soil for up to eight years.

The large size of the species, as well as its high fertility, early germination and vital growth, makes *H. sosnowskyi* a highly successful invasive species. The plant’s ability to create extensive shade has a detrimental effect on the growth and development of other plants in the surrounding areas. Consequently, only shade-tolerant species persist within the community. *H. sosnowskyi* can establish mono-species stands, altering the diversity of the forest bird community, changing the biodiversity of entire ecosystems, and transforming the landscape [15,16].

Additionally, successful Sosnowskyi hogweed outspreading can be explained by allelopathic compounds that can directly inhibit neighboring native plants or indirectly suppress native plants via the disruption of beneficial microbial mutualisms, changing the soil nematode communities or soil resources [16,17,18,19,20,21]. Allelopathy is defined as the phenomenon whereby organisms (primarily, but not exclusively, plants) release specific compounds (allelochemicals) into the environment that have an effect (positively or negatively) on the vegetation of other surrounding plants and all biotic system. Allelochemicals mainly comprise secondary and/or primary metabolites, and they are mostly water-soluble compounds that belong to a broad range of chemical compound classes.

The chemical composition of *H. sosnowskyi* raw material is diverse and contains the following components: volatile compounds/essential oils, amino acids (including glutamine), proteins, polysaccharides, tannins, carotenoids, coumarins, etc. Data related to the phytochemistry of *H. sosnowskyi* extracts are rather limited. The most thoroughly investigated are coumarins and furanocoumarins, such as imperatorin, (iso)pimpinellin, osthole, oxypeucedanin, pangelin, (iso)psoralen, methoxsalen, sphondin, (iso)bergapten and umbelliferone in hogweed extracts [22,23,24,25,26,27,28,29,,30,31,32,33]. Many of these compounds, which are found predominantly in the leaves, stems and inflorescences of *H. sosnowskyi*, are phototoxic and photocarcinogenic for both humans and animals when activated by sunlight or specific frequencies of electromagnetic waves [32,33,34,35,36,37]. A comprehensive examination of the toxicological profiles of *H. sosnowskyi* metabolites using in silico methods was recently conducted by Rassabina et al. [38].

*H. sosnowskyi* is included in the list of invasive and alien species of European Union and in the national directives of many European countries [39,40,41]; measures are being taken to control the aggressive propagation of the plant [42,43,44,45]. On other hand, despite its harmfulness, the biomass of the *H. sosnowskyi* plants could be a valuable raw biomaterial for the isolation of useful chemical compounds [38,46,47,48]. Moreover, *H. sosnowskyi* can be used in different industries, e.g., pharmaceutical, food, agrochemical, energy, construction and manufacturing [49].

It must be emphasized that the allelopathic effects of *H. sosnowskyi* on other plants have not been studied sufficiently [31,50,51,52,53,54,55], and further research studies in this area is required.

The aim of the present research is to provide detailed phytochemical analysis of aqueous extracts obtained from different plant organs (inflorescences, leaves and roots) of invasive *H. sosnowskyi*; additionally, we seek to evaluate the allelopathic properties of on the seed germination and growth of four model plants, lettuce (*Lactuca sativa* L.) and three native Lithuanian meadow herbs, perennial ryegrass (*Lolium perenne* L.), timothy (*Phleum pratense* L.) and white clover (*Trifolium repens* L.). The listed plant seeds were examined to ascertain the most allelopathically active morphological part of Sosnowsky’s hogweed. However, it is important to ascertain which of the tested plant species is the most sensitive to the secondary metabolites of *H. sosnowskyi* plants. It is evident that there is a lack of research of this kind, which is significant and could be applied in the regulation and biocontrol programs of the fast-spreading invasive weed, *H. sosnowskyi*.

## 2. Results

### 2.1. Chemical Composition of Volatile Organic Compounds (VOCs) in Heracleum sosnowskyi Extracts

Major compositional data (more than 5%) from the chemical analysis performed via GC/MS of *H. sosnowskyi* extracts obtained from various plant organs are presented in Table 1.

### 2.2. Chemical Composition of Heracleum sosnowskyi Aqueous Extracts Revealed Using the HPLC/DAD/TOF Technique

Forty-eight constituents were identified tentatively in the leaf, inflorescence and/or root *H. sosnowskyi* aqueous extracts (Table 2). All compounds were detected using DAD and TOF in the positive or negative ionization mode; some of them provided m/z ions via both (positive and negative) methods.

### 2.3. Allelopathic Effects of Heracleum sosnowskyi Aqueous Extracts on Different Plant Seeds

The effects of aqueous extracts of Sosnowsky’s hogweed roots, leaves, and inflorescences on the germination and growth of different plant seeds: lettuce (*Lactuca sativa* L.), perennial ryegrass (*Lolium perenne* L.), timothy (*Phleum pratense* L.) and white clover (*Trifolium repens* L.) were investigated. Allelopathic activity was determined at three relative concentrations of 0.1, 0.5, and 1.0. The data on the germination rate (GR) of *L. sativa*, *L. perenne*, *P. pratense* and *T. repens* seeds under the influence of aqueous extracts of *H. sosnowskyi* are presented in Table S1. The seed vigor indexes (VI) of model plants are presented in Table S2.

Sosnowsky’s hogweed aqueous root extracts have the least impact on the lettuce (*L. sativa*) seed GR (Table S1). Statistically significant differences in comparison to the control only occur at the highest relative concentration (1.0). Lettuce seed germination was suppressed by *H. sosnowskyi* root extracts by 61.17%; and GR reached 36.67 ± 10.61%. However, the *H. sosnowskyi* aqueous extracts from leaves at the 0.5 and 1.0 relative concentrations significantly reduced GR of the tested lettuce seeds, reaching only up to 55.00 ± 12.29% and 25.56 ± 10.60%, respectively (Table S1).

The GR data related to perennial ryegrass seeds indicated that Sosnowsky’s hogweed aqueous flower, leaf, and root extracts at the lowest relative concentrations (0.1) had similar effects on the seed germination rate (Table S1). GR was established from 63.33 ± 2.36% for the *H. sosnowskyi* flower extracts and up to 71.11 ± 6.29% for root extracts. The flower extracts exhibited the strongest reduction impact on GR of perennial ryegrass (*L. perenne*) seeds (Table S1). The highest tested relative concentrations, 0.5 and 1.0 suppressed seed germination by 81.02% and 93.04%, respectively. GR were determined 16.67 ± 5.93% and 6.11 ± 0.79%, in comparison to 87.78 ± 3.42% in the control group (Table S1). *H. sosnowskyi* leaf extracts also diminished ryegrass germination up to 82.27%, and GR at the highest tested concentration was only 15.56 ± 3.16% (Table S1).

Aqueous Sosnowsky’s hogweed leaf extracts had the least impact on the GR of timothy (*P. pratense*) seeds (Table S1). The highest tested concentrations, 0.5 and 1.0, suppressed timothy seed germination by 59.28% and 76.05%, respectively. GR values were 37.78 ± 5.50% and 22.22 ± 2.83% in comparison to 92.78 ± 4.16% in the control group (see Table S1). Aqueous extracts of the hogweed flowers and roots demonstrated stronger inhibitory effects. At the highest relative concentration (1.0), root extracts suppressed the germination of timothy seeds by 95.21%, with GR reaching only 4.44 ± 1.57%. At the highest relative concentration (1.0), the *H. sosnowskyi* flower extracts blocked seed germination completely, i.e., by 100% (Table S1).

Sosnowsky’s hogweed aqueous root extracts have the least impact on the clover (*T. repens*) seed GR. The lowest tested relative concentration (0.1) suppressed seed germination by 38.15% (RG = 59.44 ± 7.97%). However, for the higher concentration range (0.5 and 1.0), the inhibitory effects on clover seeds were similar: seed germination was suppressed by 68.76% and 74.57%, respectively. RG was 30.00 ± 1.36% and 24.44 ± 8.85% in comparison to 96.11 ± 5.15% in the control group (Table S1). *H. sosnowskyi* flower and leaf extracts exhibited the strongest inhibitory effects on clover seed GR. The inflorescence extracts at the highest tested concentrations (0.5 and 1.0) suppressed seed germination by 91.33% and 97.11%, respectively. GR was only 8.33 ± 1.18% and 2.78 ± 1.57, in comparison to 96.11 ± 5.15% in the control group (Table S1). The hogweed leaf extracts demonstrated the strongest inhibitory properties on clover seed germination. Even at the lowest tested concentration, the seed germination was suppressed by 52.02% (GR = 46.11 ± 14.33%). At the highest tested concentrations, 0.5 and 1.0, the hogweed leaf extracts suppressed *T. repens* seed germination by 99.42% and 100%, respectively (Table S1).

**Figure 3:**
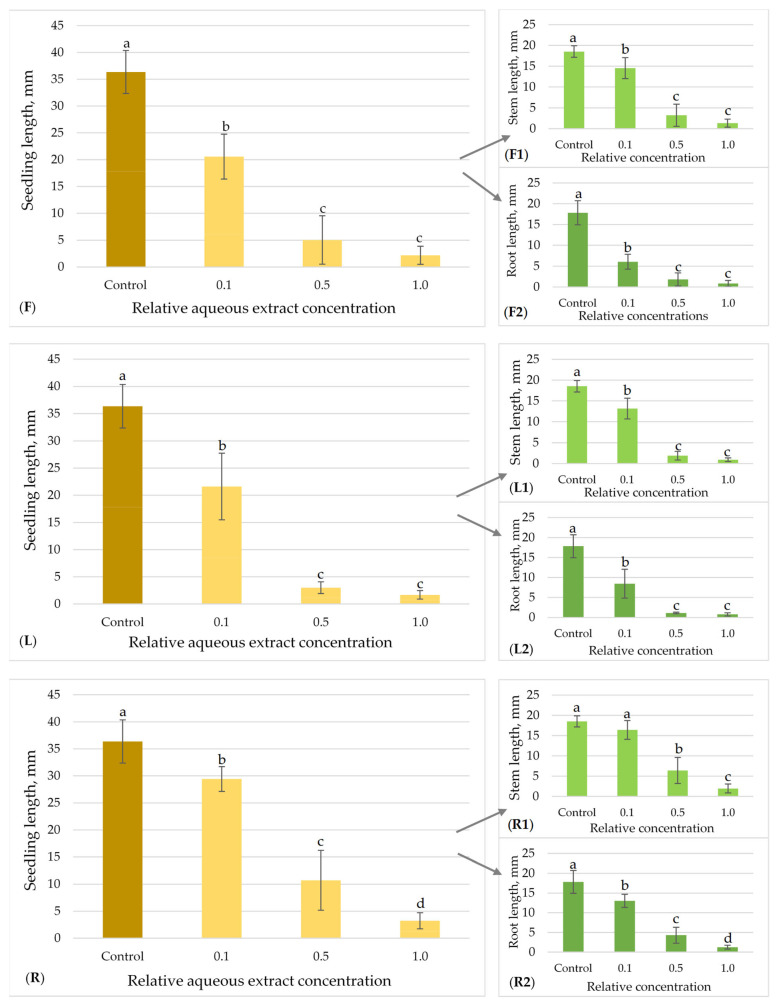
Effect of *H. sosnowskyi* aqueous extracts from flowers (**F**), leaves (**L**) and roots (**R**) on *L. sativa.* Results are expressed as the means of nine replicates (n = 180) ± SD (Standard Deviation) (bars). F, L and R demonstrate summary impact on both stem (**F1**,**L1**,**R1**) and root (**F2**,**L2**,**R2**) lengths. Different letters (a, b, c and d) above the bars indicate statistically significant differences between groups (*p* < 0.05), determined by the Kruskal–Wallis test followed by Dunn’s post hoc test.

**Figure 4:**
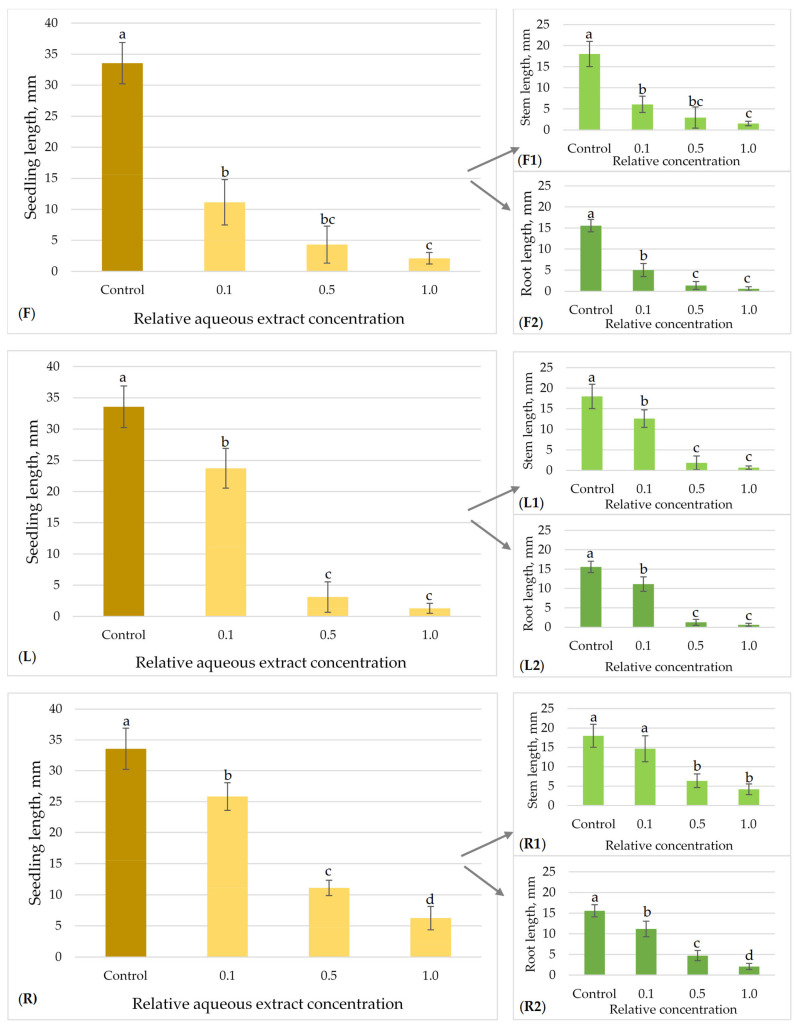
Effect of *H. sosnowskyi* aqueous extracts from flowers (**F**), leaves (**L**) and roots (**R**) *on L. perenne.* Results are expressed as the means of nine replicates (n = 180) ± SD (Standard Deviation) (bars). F, L and R demonstrate summary impact on both stem (**F1**,**L1**,**R1**) and root (**F2**,**L2**,**R2**) lengths. Different letters (a, b, c and d) above the bars indicate statistically significant differences between groups (*p* < 0.05), determined by the Kruskal–Wallis test followed by Dunn’s post hoc test.

**Figure 5:**
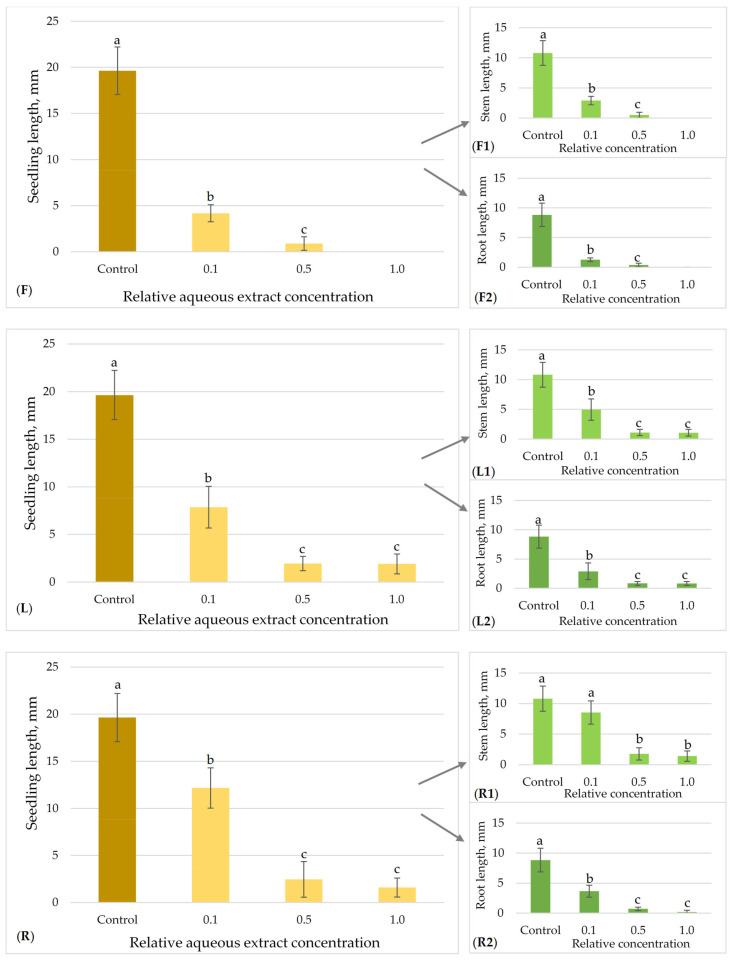
Effect of *H. sosnowskyi* aqueous extracts from flowers (**F**), leaves (**L**) and roots (**R**) on *P. pratense*. Results are expressed as the means of nine replicates (n = 180) ± SD (Standard Deviation) (bars). F, L and R demonstrate the summary impact on both stem (**F1**,**L1**,**R1**) and root (**F2**,**L2**,**R2**) lengths. Different letters (a, b and c) above the bars indicate statistically significant differences between groups (*p* < 0.05) determined by the Kruskal–Wallis test followed by Dunn’s post hoc test.

**Figure 6:**
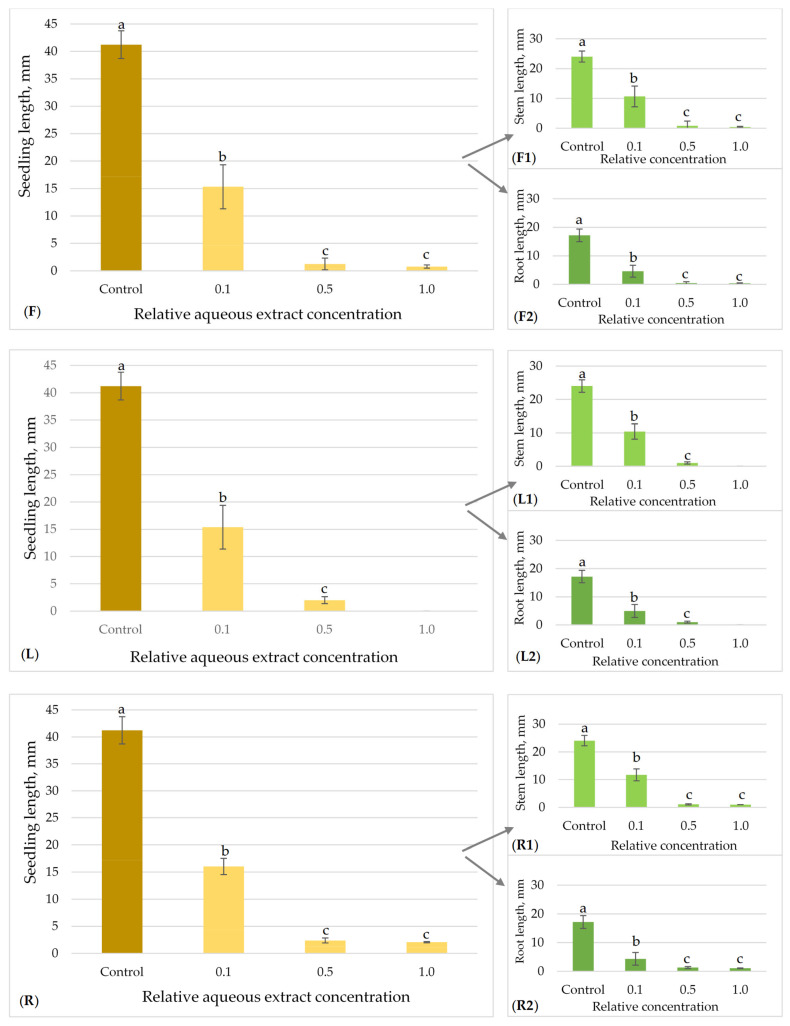
Effect of *H. sosnowskyi* aqueous extracts from flowers (**F**), leaves (**L**) and roots (**R**) *on T. repens.* Results are expressed as the means of nine replicates (n = 180) ± SD (Standard Deviation) (bars). F, L and R demonstrate the summary impact on both stem (**F1**,**L1**,**R1**) and root (**F2**,**L2**,**R2**) lengths. Different letters (a, b and c) above the bars indicate statistically significant differences between groups (*p* < 0.05), determined by the Kruskal–Wallis test followed by Dunn’s post hoc test.

The allelopathic effects of flower, leaf and root aqueous extracts of *H. sosnowskyi* on the model plant seed germination and growth are presented in Figure 3, Figure 4, Figure 5 and Figure 6.

## 3. Discussion

A more comprehensive understanding of the phytochemistry of invasive *H. sosnowskyi* plants could represent an essential factor in the search for and development of effective control methods against its aggressive spread.

The principal VOCs determined in all extracts were furan-derivatives. Chemical structures of furan-derivatives identified in *H. sosnowskyi* extracts are presented in Figure S1. The largest amounts of furanocoumarin and isopsoralen were determined in the flower and leaf extracts (an average 55.4 and 47.3%, respectively) (Table 1). The root extracts contained notable quantities of pimpinellin (44.9 ± 5.1%), isopimpinellin (average 14.9%), bergapten (12.4%), isopsoralen (9.1%) and isobergapten (8.0%). The inflorescence extracts were characterized by isopsoralen (55.4 ± 4.3%), methoxsalen (average 14.7) and pimpinellin (13.8%) (Table 1). The leaf extracts comprised the major volatile constituents, such as isopsoralen (47.3 ± 3.1%), methoxsalen (average 30.1%) and bergapten (11.2%) (Table 1). Additionally, minor quantities (less than 5%) of some compounds, such as terpenoids, phenols, alcohols, acids, etc., were determined among the volatiles in the extracts. The majority of the VOCs found in this study have previously been reported in other studies related to the phytochemistry of essential oils and other extracts of *H. sosnowskyi* and other *Heracleum* species [24,25,26,29,30,31,33,37,38,47,53,56,57]. In accordance with the findings of this study, in many cases, furanocoumarins were found to be the predominant compound class among the volatiles of *H. sosnowskyi* [24,26,30,31,32,33,34,35,36,38]. It is rightly assumed that furanocoumarins synthesized by *H. sosnowskyi* are the most bioactive/toxic compounds among the other metabolites of the plant.

A total of 38 fatty acids (including suberic, stearic, oleic, phenyloctanoic, linoleic, a-linolenic, etc.) were identified in the extracts obtained from various *H. sosnowskyi* biomass parts using dichloromethane (the most effective solvent for lipid extraction) in the study conducted by Borska et al. [53]. Not using any specific extraction conditions allowed us to identify several fatty acids, including linolenic and glutaric acids, which were found in all extracts, and phenyl octanoic, suberic and oleic acids, which were found in the inflorescences (Table 2). *p*-Anisic (4-methoxybenzoic) acid was identified tentatively by us in the aqueous inflorescence extracts (Table 2), while it was determined in the leaves and roots of Sosnowskyi hogweed from Latvia [53]. A significant number of acids identified in this study have also been detected in *H. sphondylium* and *H. brunonis* [56,58].

In contrast to the results obtained in the present study (Table 2), a considerable number of esters have been identified in earlier studies of *H. sosnowskyi* [25,26,29,30,38,53].

In addition to monoterpenes and sesquiterpenes, neophytadiene, a diterpene, was identified in the flower extracts using the HPLC/DAD/TOF technique (Table 2). This constituent has previously been determined in Sosnowskyi hogweed from Latvia [53].

To the best of our knowledge, the already-published data on the synthesis of flavonoids by *H. sosnowskyi* are very limited. Kaempferol derivatives, such as astragalin and nicotiflorin (kaempferol 3-*O*-glucoside and kaempferol-3-*O*-rutinoside, respectively) were identified in leaf and inflorescence extracts in the present study (Table 2), while kaempferol was found in the hogweed leaves from Poland [34]. The chemical structures of flavonoids identified in the *H. sosnowskyi* extracts under study are shown in Figure S2. Furanocoumarins present in our extracts (Table 2, Figure S3), candibirin F and candinols (A and B), have previously been isolated from the roots of *H. candicans* [56,59,60], while apterin was identified in *H. dissectum* and *H. platytaenium* [56,61,62]. Coumarin, umbelliferon was found in both flower and root extracts (Table 2, Figure S3); however, this constituent was only found in the flowers of *H. sosnowskyi* of Latvian origin [53] and other species of *Heracleum* [38,57]. As demonstrated in Table 2, other coumarins, limettin and yunngnins (A and B), identified tentatively in the inflorescences and roots, have been determined previously in other *Heracleum* species, including *H. mantegazzianum* and *H. yunngningense* [56,63,64]. In addition, furanocoumarin, hermandiol was isolated from the same *H. yunngningense* species [64]. The chemical structures of all furan-derivatives and coumarins identified in the *H. sosnowskyi* extracts under consideration are presented in Figure S3.

The phytochemical analysis revealed that *H. sosnowskyi,* like other invasive plants, is distinguished by its diverse chemical composition, which is mainly characterized by furanocoumarins. This facilitates its ability to adapt to a variety of environmental conditions and compete successfully with native species.

A key aim of this study was to ascertain the allelopathic impact of Sosnowsky’s hogweed aqueous extracts on other plant species. As demonstrated in the relevant literature, meadows and grasslands are the habitats most strongly affected by this invasion [8,10,11,40]. For the purpose of this investigation, three native meadow plant species were selected: perennial ryegrass (*L. perenne*), timothy grass (*P. pretense*), and white clover (*T. repens*). Additionally, lettuce (*L. sativa*) was selected as the most widely used model plant species.

The allelopathic properties of flower, leaf, and root aqueous extracts of Sosnowsky‘s hogweed on lettuce (*L. sativa)* seedlings growth are presented in Figure 3. The mean length of the control lettuce seedlings was 36.36 ± 4.01 mm. Significant differences were identified among all of the tested extracts and concentrations (0.1; 0.5; 1.0) in relation to lettuce growth in comparison to the control (Kruskal–Wallis, Dunn’s post hoc test (*p* < 0.05) (Figure 3). The most significant suppression of lettuce seedling growth was observed for *H. sosnowskyi* leaf aqueous extracts. The model plant seedlings exhibited a 40.57% decrease in growth (from 36.36 ± 4.01 mm to 21.61 ± 6.13 mm) at 0.1, a 91.72% (to 3.01 ± 1.08 mm) at 0.5, and a 95.35% decrease (to 1.69 ± 0.79 mm) at 1.0 relative concentration, compared to the control (Figure 3L). In a case of *H. sosnowskyi* flower extracts, we can see similar effects (Figure 3F). Lettuce seedling growth decreased by 86.17% (from 36.36 ± 4.01 mm to 5.03 ± 4.51 mm) at 0.5, and by 94.00% (to 2.18 ± 1.70 mm) at 1.0 relative concentration compared to the control (Figure 3F). In addition, it was observed that *H. sosnowskyi* leaf and flower extracts demonstrated a greater inhibitory effect on the root growth as compared to stem growth in seedling samples (Figure 3(L1,L2,F1,F2)).

The allelopathic effects of Sosnovsky hogweed flower, leaf, and root aqueous extracts on perennial ryegrass seedlings growth are presented in Figure 4. The mean length of the control seedlings was 33.56 ± 3.32 mm. All of the tested extracts indicated statistically significant differences between the effects at all concentrations (0.1; 0.5; 1.0) on ryegrass growth, in comparison to the control (Figure 4). The most significant suppression of perennial ryegrass seedlings’ growth was observed in aqueous extracts of *H. sosnowskyi* flowers (Figure 4F) and leaves (Figure 4L). The lowest concentration tested of flower extracts (0.1) suppressed seedling growth by 66.84% (from 33.56 ± 3.32 mm to 11.13 ± 3.66 mm). The strongest inhibitory effects were exhibited at higher tested concentrations: ryegrass seedlings exhibited an 87.16% decrease in growth (4.31 ± 2.99 mm) at 0.5, and a 93.74% decrease (2.10 ± 0.93 mm) at 1.0 relative concentration, compared to the control (Figure 4F). In the case of *H. sosnowskyi* leaf extracts, ryegrass seedlings demonstrated a significant reduction in growth of 29.32% (from 33.56 ± 3.32 mm to 23.72 ± 3.19 mm) at 0.1, a 90.76% decrease (to 3.10 ± 2.45 mm) at 0.5, and a 96.16% decrease (to 1.29 ± 0.79 mm) at 1.0 relative concentration compared to the control (Figure 4L). The hogweed root extracts exhibited a lesser inhibitory effect at the highest concentration range (0.5 and 1.0): ryegrass seedling growth diminished by 66.93% (from 33.56 ± 3.32 mm to 11.10 ± 1.23 mm) at 0.5, and by 81.38% (to 6.25 ± 1.89 mm) at the 1.0 relative concentration, compared to the control (Figure 4R). In addition, it was revealed that the highest tested concentrations of the hogweed flower extract (0.5 and 1.0) demonstrated a greater inhibitory effect on root growth as compared to stem growth in perennial ryegrass seedling samples (Figure 4(F1,F2)).

The allelopathic effects of flower, leaf, and root aqueous extracts of Sosnowsky’s hogweed on timothy seedlings growth are presented in Figure 5. The mean length of the control seedlings was 19.64 ± 2.57 mm. All the tested *H. sosnowskyi* extracts showed statistically significant differences between the effects of all tested concentrations (0.1; 0.5; 1.0) on timothy seed growth in comparison to the control (Figure 5). The most significant suppression of timothy seedling growth was observed in the *H. sosnowskyi* flower aqueous extracts. The model plant seedlings exhibited a 78.77% decrease in growth (from 19.64 ± 2.57 mm to 4.17 ± 0.92 mm) at 0.1, a 95.47% diminishing (to 0.89 ± 0.73 mm) at 0.5, and a 100% decrease (seed germination was completely inhibited) at 1.0 relative concentration compared to the control (Figure 5F). Following treatment with hogweed leaf extracts, the growth of timothy seedlings decreased by 59.98% (from 19.64 ± 2.57 mm to 7.86 ± 2.19 mm) at a relative concentration of 0.1, by 90.12% (to 1.94 ± 0.75 mm) at a concentration of 0.5, and by 90.33% (to 1.90 ± 1.05 mm) at a relative concentration of 1.0, compared to the control (Figure 5L). The *H. sosnowskyi* root extracts exhibited a high inhibitory effect within the 0.5–1.0 relative concentration range on timothy seedling growth, decreasing by 87.48% (to 2.46 ± 1.90 mm) and 91.90% (to 1.59 ± 1.01 mm), respectively (Figure 5R). In addition, the highest tested concentrations of the root extracts (0.5 and 1.0) demonstrated a greater inhibitory effect on timothy’s seedling root growth (Figure 5(R1,R2)).

The allelopathic properties of flower, leaf, and root aqueous extracts of Sosnowsky‘s hogweed on clover seedlings growth are presented in Figure 6. The mean length of the control seedlings was 41.22 ± 2.53 mm. All the tested hogweed extracts showed statistically significant differences between the effects at all tested concentrations (0.1; 0.5; 1.0) on clover growth, in comparison to the control (Figure 6). Sosnowsky’s hogweed aqueous flower, leaf, and root extracts at the lowest relative concentrations (0.1) had similar effects on clover seedlings, and they exhibited a 61.09% to 62.83% decrease in growth in comparison to the control group (Figure 6F,L,R). At a concentration 0.5, hogweed inflorescence extracts demonstrated the strongest allelopathic effects: clover seedling growth decreased by 96.99% (from 41.22 ± 2.53 mm to 1.24 ± 1.06 mm). The hogweed leaf and root extracts (at a 0.5 relative concentration) exhibited similar suppressing effects, with seedling growth decreasing by 94.66% and 94.23%, respectively. At the highest concentrations (1.0) of Sosnowsky‘s hogweed leaves, clover seedling growth was blocked completely, i.e., by 100%. Specifically, the measurements revealed 98.11% (from 41.22 ± 2.53 mm to 0.78 ± 0.31 mm) and 94.98% (from 41.22 ± 2.53 mm to 2.07 ± 0.11 mm) decreases in clover seedling length in the case of tests with *H. sosnowskyi* flower and root extracts.

Methoxsalen, bergapten and angelicin (isopsoralen) are identified as the predominant furanocoumarins in Sosnowsky’s hogweed, with documented evidence of their allelopathic properties [31,38,52]. The inhibitory activity of angelicin present in *H. sosnowskyi* fruit extracts was found to be the highest compared with the other furocoumarins tested on monocots (*Lolium multiflorum*, *Phleum pratensis*, *Festuca pratesis*, *Lolium perenne*) and dicots (*Tripholium repens*, *Trifolium pretense*) [31]. In the study conducted by Mishyna et al., *H. sosnowskyi* fruits incorporated in the soil increased concentrations of octanol, octanal and octyl acetate in the soil; a positive correlation was evaluated between the octanol concentration in the soil and the plant growth inhibition of *L. sativa*, *T. repens* and *L. multiflorum* seedlings, and the radicle growth inhibition of *L. sativa* and *L. multiflorum* seedlings [52]. A furanocoumarin, psoralen (ficusin) present in *H. sosnowskyi* lacks strong evidence as a proven allelochemical compared to others such as methoxsalen or angelicin; its role appears secondary to more active furanocoumarins [38,65]. No bioassays demonstrate pimpinellin or isopimpinellin’s specific inhibition of seed germination or seedling growth in competing species, while the toxicity and other pharmacological properties of these compounds are thoroughly documented [26,29,33,56,58,66,67,68]. *H. sosnowskyi* essential oil, containing major compounds such as octyl acetate, hexyl 2-methylbutanoate and octanol, demonstrated the strongest herbicidal effects against *Bromus secalinus* and *Avena fatua* [25]. Aldehydes in the hogweed seed extracts are also associated with allelopathic potential [38]. Lipids (mainly fatty acids) of *H. sosnowskyi* demonstrated strong toxic effects with prominent allelopathic potential [53].

The results obtained demonstrate the significant inhibitory effects of Sosnowsky’s hogweed aqueous extracts, especially flower and leaf extracts, which resulted in the most significant suppression of germination and growth in all the tested plant species.

## 4. Materials and Methods

### 4.1. Plant Material

The raw plant material of *H. sosnowskyi* (up to 2.5–3.0 kg) was collected in full blooming stage (in July 2023) from an abandoned field, on the forest edge (Southern Lithuania, Alytus district, Daugai village (coordinates: 54°21′41.0″ N 24°17′42.6″ E). The Sosnowsky’s hogweed thicket covered an area of up to 450 m^2^ and had been established there for over ten years. The collection of the entire plants was conducted in a randomized manner, and the material was subsequently divided into three distinct parts: roots, leaves, and inflorescences. The raw material (comprising above- and below-ground parts) was transferred immediately to the laboratory and subjected to a drying process at a room temperature (20–25 °C) under conditions that ensured shade and adequate ventilation for a period of 3 weeks. The separation of the leaves, inflorescences and roots was conducted prior to the drying process.

Four plant species were selected as model plants for the allelopathy test: lettuce (*Lactuca sativa*), perennial ryegrass (*Lolium perenne*), timothy (*Phleum pratense*) and white clover (*Trifolium repens*). Lettuce seeds were chosen because this model plant is one of the most commonly used in allelopathy studies. The selection of the three remaining plant species was based on two key factors: first, their abundance in abandoned agricultural fields and meadows, where Sosnowsky’s hogweed is most commonly found; second, the lack of scientific data on the allelopathic effects of the invasive plant on these native perennial species. The seeds of the plant specimens were obtained from local markets specializing in the sale of horticulture produce.

### 4.2. Preparation of H. sosnowskyi Aqueous Extracts for HPLC/DAD/TOF Analysis and Allelopathic Tests

Samples of fully air-dried of *H. sosnowskyi* inflorescences, leaves and roots were ground separately into a homogenous powder and protected from light and humidity until the analyses were conducted. Infusions were made of 15 g of crushed raw material and 250 mL of deionized water (0.06 g/mL, weight of dried herbal matter), covered tightly with aluminum foil, and left to settle at 20 °C for 24 h. After soaking, the mixtures were filtered through an 11 μm pore size filter paper (Whatman, GmbH, Germany) to remove the solid plant material from the liquid. Afterwards, the aqueous extracts were used for HPLC/DAD/TOF analysis and allelopathic assay.

### 4.3. Preparation of H. sosnowskyi Extracts for Analysis of Volatile Organic Compounds (VOCs)

A mixture of diethyl ether and hexane (1:1, Vol:Vol) was used for the extraction of VOCs from aqueous solutions (prepared according to the method described in Section 4.2). Around 60 mL of each aqueous solution was extracted with 6 mL of the organic mixture. The extraction procedure was performed in an ultrasonic bath at room temperature (21–24 °C) for a period of 30 min for leaves and inflorescences and 1 h for roots. Diethyl ether was purchased from C. Roth GmbH & Co. (Karlsruhe, Germany). Hexane (of HPLC grade) was from Honeywell (Seelze, Hanover, Germany).

### 4.4. GC-MS Analysis

Analyses were performed on a chromatograph Agilent 6890 N GC system (Agilent Technologies Inc., Santa Clara, CA, USA) interfaced to a HP 5973 Mass Selective Detector (Agilent Technologies/HP) and fitted with a capillary column DB-5MS ((5%-phenyl)-methylpolysiloxane, 30 m × 0.32 mm i.d., film thickness 0.25 µm) (Agilent, J&W Scientific, Santa Clara, CA, USA). The GC oven’s temperature was programmed as follows: increased from 50 °C (isothermal for 1 min) to 250 °C at a rate of 10 °C/min, and then the final temperature was maintained for 5 min. The injection volume was 2 or 5 µL. The temperature of the injector was 240 °C. The flow rate of the carrier gas (helium) was 1 mL/min, split 1:20. At least 3 repetitions (n ≥ 3) per analysis were performed. The temperature of the ion source was 230 °C. Mass spectra in electron mode were generated at 70 eV, 3.10 scans/s, mass range 33–500 m/z.

The percentage composition of the VOCs was computed from GC peak areas without correction factors. The qualitative analysis was based on co-injection of some reference terpenoids and C_8_-C_28_ n-alkane series and computer mass spectra libraries (Flavour and Fragrance of Natural and Synthetic Compounds 2 (FFNSC 2), Wiley and NIST). Identification was approved when the computer match with mass spectral libraries had probabilities above 90%.

### 4.5. HPLC-DAD-MS (TOF) Analysis

Aqueous extracts of *H. sosnowskyi* inflorescences, leaves and roots were analyzed via the HPLC technique using a system HPLC/Diode Array Detector (DAD)/Time of Flight (TOF) (Agilent 1260 Infinity (Agilent Technologies, Waldbronn, Germany) and Agilent 6224 TOF (Agilent Technologies, Santa Clara, CA, USA)) equipped with a reverse phase column ZORBAX Eclipse XDB (C18, size of particles 5 µm particle size, column parameters 150 × 4.6 mm (Agilent Technologies, Santa Clara, CA, USA)). The column temperature was maintained at 25 °C during the chromatographic analysis. The solvents used for the procedure were as follows: A—deionized water containing 2% of acetic acid and 10% of acetonitrile, and B—methanol. Acetonitrile and methanol were purchased from Honeywell (Seelze, Hanover, Germany), and acetic acid—from Sigma Aldrich Co. (St. Louis, MO, USA). The following stepwise gradient elution method was applied in the HPLC system: the initial composition was 95% (A)/5% (B); from 0 to 4 min, the ratio changed from the initial ratio to 100% (A)/0% (B); from 4 to 15 min, the ratio 0% (A)/100% (B) was achieved and, then, from 15 to 20 min, the isocratic mode was maintained; from 20 to 25 min, the ratio changed to 100% (A)/0% (B); and, finally, for the last 5 min, the initial composition (95% (A)/5% (B)) was achieved. The chromatographic separation was performed at a flow rate of 1.0 mL/min. Molecules were ionized in positive and negative modes using the Electrospray Ionization Interface (ESI). Extract volumes ranging from 5 to 10 µL were injected using an automated sampler. The TOF acquisition parameters were set at mass range 50–1500 m/z. Ionization source conditions were as follows: nebulizer 30 psig, drying gas temperature 350 °C, drying gas flow rate 30 L/min and fragmentation voltage 150 V. An internal calibration solution with known reference masses was applied to achieve the mass accuracy of the recorded data. The DAD range was set from 190 to 600 nm with selected scans at 230, 250, 260, 286, 310 and 410 nm.

### 4.6. Seed Germination Bioassay

Tested plant seeds were surface sterilized with 70% ethyl alcohol for exactly 2 min and then thoroughly washed three times with deionized water. Subsequently, 20 seeds per glass Petri dish (Ø 9 cm) were placed on Whatman filter paper, and 1.5 mL of the test or control solution was added. The seeds were stored under conditions of darkness and at a temperature of 22 °C, using a Fitotron thermostat (Weiss Umwelttechnik GmbH, Germany) for a period of five days. The number of germinated seeds was counted after 5 days of incubation. A seed was considered to have germinated when the seed coat had broken and the radicle emerged.

A quantity of 1.5 mL of the solution (0.06 mg/mL, (dry herbal weight/water volume)), prepared according a procedure described in 4.2.), was utilized for the allelopathic tests; it was designated as 1.0 of relative concentration. Deionized water was used in the control plates. Images of control plates of the tested plants seedlings (a—lettuce (*Lactuca sativa* L.), b—perennial ryegrass (*Lolium perenne* L.), c—timothy (*Phleum pratense* L.) and d—white clover (*Trifolium repens* L.)) are given in Figure S4.

Calculation equations of the germination rate (GR) and vigor index (VI) are presented in Table S3.

### 4.7. Statistical Data Analyses

Statistical analyses were conducted using PAST version 4.03 (LO4D, Seattle, WA, USA) to evaluate data reliability and detect significant differences. Data normality was assessed using the Shapiro–Wilk test with a significance level of α = 0.05. As the data were not normally distributed, differences among independent groups were analyzed using the Kruskal–Wallis test (α = 0.05), followed by Dunn’s post hoc test (α = 0.05) for multiple comparisons. Pairwise comparisons between groups were further examined using the Mann–Whitney test to assess statistically significant differences in germination rate. Statistical significance was set at *p* ≤ 0.05.

## 5. Conclusions

The characterization of the chemical profile of an aggressive invasive *H. sosnowskyi* plant, as well as the identification of chemical phenotypes, has the potential to support weed biocontrol methods and to improve agent establishment rates and overall impacts on target weeds. Due to their diverse chemical composition, invasive plants can easily adapt to various environmental conditions and successfully compete with native species. The results obtained demonstrated the remarkable inhibitory effects of all Sosnowsky’s hogweed aqueous extracts; significant differences were revealed in most cases. In general, the application of flower and leaf extracts resulted in the most notable suppression of germination and growth in all the tested plant species. For the first time, the seeds of some native meadow plants were applied as a model herbal material for *H. sosnowskyi* allelopathic tests. The plants most sensitive to *H. sosnowskyi* aqueous extracts were considered to be seeds of white clover (*Trifolium repens* L.) and ryegrass (*Lolium perenne* L.). *H. sosnowskyi* leaf extracts had stronger inhibition effects on seed germination and growth of lettuce (*Lactuca sativa* L.) and clover; the same was true for flower extracts on ryegrass, clover and timothy (*Phleum pratense* L.). Sosnowsky’s hogweed root extracts exhibited a weaker inhibitory impact on the germination rate and growth of the seeds. These differences could be explained by the chemical variability of various *H. sosnowskyi* aqueous extracts. Indeed, the highest concentrations of furanocoumarins were determined in flower and leaf extracts, with isopsoralen being particularly prevalent. However, a wider variety of furanocoumarins was detected in the root extracts, albeit in smaller quantities. It is also noteworthy that the predominant furanocoumarin present in root extracts was identified as pimpinellin, while isopsoralen was detected at concentrations several times lower than in above-ground parts. It is not possible to claim that only individual main constituents or compound classes are responsible for the allelopathic effects of the *H. sosnowskyi* aqueous extracts; rather, it can be posited that the whole multicomponent complex is active. Because of the phenomenon of synergism, the impact of not only major but also minor compounds must not be neglected.

This study addresses the gaps in research related to the phytochemistry of secondary metabolites and allelopathy of Sosnowsky’s hogweed, thus contributing significantly to the advancement of knowledge in this field. The study revealed the importance of investigating allelopathic mechanisms, improving our understanding of their role in the spread of invasive species. To control the aggressive and invasive *H. sosnowskyi* species, we recommend annihilating it before the flowering stage and ensuring that the removed biomass is not left in territories colonized by these plants.

## Figures and Tables

**Table 1:** Percentage of main volatile organic compounds (≥5%) determined in *H. sosnowskyi* inflorescence, leaf and root extracts (n = 3, average ± SD (Standard Deviation)).

No.	Compound	RI	Flowers	Leaves	Roots
1.	Isopsoralen (2H-Furo[2,3-h]chromen-2-one; Furo(2,3-h)coumarin) (syn. Angelecin)	1810	55.4 ± 4.3	47.3 ± 3.1	9.1 ± 2.0
2.	Ficusin (7H-Furo[3,2-g][1]benzopyran-7-one; Furo[4′,5′:6,7]coumarin; 7H-Furo[3,2-g]chromen-7-one) (syn. Psoralen)	1842	3.6 ± 1.0	5.3 ± 1.7	1.9 ± 0.8
3.	Isobergapten (2H-Furo[2,3-h]-1-benzopyran-2-one, 5-methoxy; 5-Methoxy-2H-furo[2,3-H]chromen-2-one)	2016	1.7 ± 0.4	0.1 ± 0.1	8.0 ± 2.2
4.	Methoxsalen (7H-Furo[3,2-g][1]benzopyran-7-one; 9-Methoxy-7H-furo[3,2-g]chromen-7-one) (syn. Ammoidin; Meladinin; Meloxine; Oxsoralen; Xanthotoxin)	2032	14.7 ± 4.3	30.1 ± 5.8	3.7 ± 0.9
5.	Bergapten (7H-Furo[3,2-g][1]benzopyran-7-one, 4-methoxy; 5-Methoxypsoralen) (syn. Bergaptan; Heraclin)	2056	6.6 ± 2.5	11.2 ± 3.3	12.4 ± 3.3
6.	Pimpinellin (5,6-Dimethoxy-2H-furo[2,3-H]chromen-2-one; 2H-Furo[2,3-h]-1-benzopyran-2-one, 5,6-dimethoxy)	2118	13.8 ± 4.0	0.2 ± 0.1	44.9 ± 5.1
7.	Isopimpinellin (7H-Furo[3,2-g][1]benzopyran-7-one, 4,9-dimethoxy; 4,9-Dimethoxy-7H-furo[3,2-g]chromen-7-one) (syn. 5,8-Dimethoxypsoralen; 5,8-Dimethoxy-6,7- furanocoumarin)	2248	1.2 ± 0.3	2.9 ± 0.0	14.9 ± 1.7

RI—Retention index on non-polar column DB-5MS.

**Table 2:** Tentative identification of main compounds in aqueous *Heracleum sosnowskyi* inflorescence, leaf and root extracts analyzed by HPLC-DAD-TOF.

No.	Compound	Formula	Molecular Weight	m/z ESI^+^ (Da)	m/z ESI^−^ (Da)
	Acids				
1.	Glutaric acid ^L,F,R^	C_5_H_8_O_4_	132.11	132.100	
2.	*p*-Anisic (4-Methoxybenzoic) acid ^F^	C_8_H_8_O_3_	152.15		151.004
3.	Suberic (Octanedioic) acid ^F^	C_8_H_14_O_4_	174.19		173.083
4.	Quinic acid ^L,F^	C_7_H_12_O_6_	192.17		191.020
5.	Phenyloctanoic acid ^F^	C_14_H_20_O_2_	220.31	221.049	
6.	Linolenic acid (9,12,15-Octadecatrienoic) acid ^L,F,R^	C_18_H_30_O_2_	278.45	279.157	
7.	Oleic (*cis*-9-Octadecenoic) acid ^F^	C_18_H_34_O_2_	282.47		281.124
8.	Chlorogenic (caffeoylquinic) acid ^L,R^	C_16_H_18_O_9_	354.31	355.099	353.086
9.	3-*O*-Feruloylquinic acid ^L^	C_17_H_20_O_9_	368.31		367.101
10.	Isochlorogenic (3,4-dicaffeoylquinic) acid ^L,R^	C_25_H_24_O_12_	516.45		515.139
11.	Chrysophanic acid (Chrysophanol; 1,8-Dihydroxy-3-methylanthraquinone) ^R^	C_15_H_10_O_4_	254.24		253.143
12.	Gelseminic (Chrysatropic) acid ^R^ (Scopoletin; 7-Hydroxy-6-methoxy-2H-chromen-2-one)	C_10_H_8_O_4_	192.16		191.018
13.	Indole-3-acetyl-L-aspartic acid (Indoleacetylaspartate) ^F^	C_14_H_14_N_2_O_5_	290.27		289.034
	Esters				
14.	Isoamyl butyrate ^L,F^	C_9_H_18_O_2_	158.24	158.995	
15.	Hexyl hexanoate ^L^	C_12_H_24_O_2_	200.32	199.097	
	Terpenoids				
16–19.	Monoterpenes (Limonene, Ocimene, Terpinene or Terpinolene) ^L^	C_10_H_16_	136.24	135.030	135.019
20.	Germacrene D ^L,F^	C_15_H_24_	204.35	205.095	
21.	Neophytadiene ^F^	C_20_H_38_	278.52	279.036	277.033
	Flavonoids				
22.	Quercetin 7,3,4-trimethyl ether ^L,F,R^	C_18_H_16_O_7_	344.3		343.035
23.	Astragalin (Kaempferol 3-*O*-glucoside) ^L,F^	C_21_H_20_O_11_	448.38	449.105	447.091
24.	Baicalin (7-D-Glucuronic acid-5,6-dihydroxyflavone) ^R^	C_21_H_18_O_11_	446.36	447.120	
25.	Nicotiflorin (Kaempferol-3-*O*-rutinoside) ^L,F^	C_27_H_30_O_15_	594.50	595.159	593.148
26.	Rutin (Quercetin-3-*O*-rutinoside) ^L,F^	C_27_H_30_O_16_	610.52	611.155	609.145
	Furano-derivatives				
27.	Coumaran ^L,F,R^	C_8_H_8_O	120.15	121.050	
28.	5,6,7,7a-Tetrahydro-4,4,7a-trimethyl-2(4h)-benzofuran ^L,F,R^	C_11_H_18_O	166.26	166.085	165.055
29.	Isopsoralen ^L,F,R^	C_11_H_6_O_3_	186.16	187.038	
30.	Ficusin ^L,F,R^	C_11_H_6_O_3_	186.16	187.098	
31.	Methoxsalen ^F,R^	C_12_H_8_O_4_	216.19	217.046	
32. 33.	Bergapten/Isobergapten ^L,F,R^	C_12_H_8_O_4_	216.19	217.002	
34. 35.	Pimpinellin/Isopimpinellin ^L,F,R^	C_13_H_10_O_5_	246.21	247.058	
35.	Imperatorin ^F,R^	C_16_H_14_O_4_	270.28	271.225	
37.	L-Arabinofuranose ^L^	C_5_H_10_O_5_	150.13	149.109	
38.	Bakuchicin (Furo[2,3-f]chromen-7-one; 7H-Furo(2,3-f)(1)benzopyran-7-one) ^R^	C_11_H_6_O_3_	186.16	187.037	
39.	Hermandiol ((8S)-8-(1,2-dihydroxypropan-2-yl)-8,9-dihydrofuro[2,3-h]chromen-2-one) ^R^	C_14_H_14_O_5_	262.26	263.058	
40.	Byakangelicol (9-[[(2R)-3,3-dimethyloxiran-2-yl]methoxy]-4-methoxyfuro[3,2-g]chromen-7-one) ^F^	C_17_H_16_O_6_	316.30	315.071	
41.	Apterin ^F^	C_20_H_24_O_10_	424.40		423.067
42.	Candinol A ^R^	C_21_H_16_O_6_	364.35	365.133	
43.	Candinol C ^R^	C_32_H_32_O_12_	608.60		607.102
44.	Candibirin F ^R^	C_34_H_34_O_11_	618.64		617.187
	Coumarins				
45.	Limettin (5,7-Dimethoxycoumarin) ^F^	C_11_H_10_O_4_	206.19		205.050
46.	Umbelliferone (7-Hydroxycoumarin; 7-Hydroxy-2H-chromen-2-one) ^F,R^	C_9_H_6_O_3_	162.14		161.024
47.	Yunngnin A ^R^	C_12_H_10_O_6_	250.05		248.956
48.	Yunngnin B ^R^	C_11_H_8_O_5_	220.04	221.048	

^L,F,R^ Presence of compounds in leaf (L), inflorescence (F) and root (R) extracts.

## Data Availability

Data are contained within the article and Supplementary Materials.

## Supplementary Materials

The following supporting information can be downloaded at: https://www.mdpi.com/article/10.3390/plants15030346/s1, Figure S1: Chemical structures of furan derivatives: 1—isopsoralen; 2—ficusin; 3—isobergapten; 4—methoxsalen; 5—bergapten; 6—pimpinellin and 7—isopimpinellin, identified in *H. sosnowskyi* extracts. Structures were drawn using the ChemDraw (version 16.0) molecule editor. Numbering of compounds (1–7) is identical to the numbers indicated in Table 1; Figure S2: Chemical structures of flavonoids: 22—quercetin 7,3,4-trimethyl ether; 23—astragalin; 24—baicalin; 25—kaempferol-3-O-rutinoside and 26—rutin, identified in *H. sosnowskyi* extracts; Figure S3: Chemical structures of furan derivatives (37—L-arabinofuranose; 38—bakuchicin; 39—hermandiol; 40—byakangelicol; 41—apterin; 42—candinol A, 43—candinol C and 44—candibirin F) and coumarins (45—limettin; 46—umbelliferone; 47—yunngnin A and 48—yunngnin B), identified in *H. sosnowskyi* extracts; Figure S4: Photos of control plates of the tested plants seedlings: a—lettuce (*Lactuca sativa* L.), b—perennial ryegrass (Lolium perenne L.), c—timothy (*Phleum pratense* L.) and d—white clover (*Trifolium repens* L.); Table S1. Inhibitory effects of aqueous extracts of *H. sosnowskyi* inflorescences, leaves and roots on the germination rate (GR, %) of *L. sativa*, *L. perenne*, *P. pratense* and *T. repens* seeds. Data are presented as the means of nine treatments (n = 180) ± SD (Standard Deviation). Different letters (a, b, c and d) with the germination rate values indicate statistically significant differences between groups (*p* < 0.05), determined by the Kruskal–Wallis test followed by the Mann-Whitney pairwise test; Table S2: Seed vigor index (VI) of lettuce (*Lactuca sativa* L.), perennial ryegrass (*Lolium perenne* L.), timothy (*Phleum pratense* L.) and white clover (*Trifolium repens* L.); Table S3: Calculation of germination rate (GR) and vigor index (VI).

## Abbreviations

The following abbreviations are used in this manuscript:

GC/MS	Gas Chromatography/Mass Spectrometry
HPLC/DAD/TOF	High Performance Liquid Chromatography/Diode Array Detection/Time-of-Flight-Mass Spectrometry
VOC	Volatile Organic Compounds
GR	Germination Rate
VI	Vigor index

## References

[1] World Flora Online WFO Plant List. https://www.worldfloraonline.org.

[2] Plants of the World Online. Kew, Royal Botanical Gardens. https://powo.science.kew.org.

[3] Jahodová Š., Fröberg L., Pyšek P., Geltman D., Trybush S., Karp A., Pyšek P., Cock M.J.W., Nentwig W., Ravn H.P. (2007). Taxonomy, identification, genetic relationships and distribution of large *Heracleum* species in Europe. Ecology and Management of Giant Hogweed (Heracleum mantegazzianum).

[4] Jakubowicz O., Żaba C., Nowak G., Jarmuda S., Żaba R., Marcinkowski J.T. (2012). *Heracleum Sosnowskyi* Manden. Ann. Agric. Environ. Med..

[5] Jahodová Š., Trybush S., Pyšek P., Wade M., Karp A. (2007). Invasive species of *Heracleum* in Europe: An insight into genetic relationships and invasion history. Divers. Distrib..

[6] Booy O., Cock M., Eckstein L., Hansen S.O., Hattendorf J., Hüls J., Jahodová Š., Krinke L., Moravcová L., Müllerová J., Nielsen C., Ravn H.P., Nentwig W., Wade P.M. (2005). The Giant Hogweed Best Practice Manual. Guidelines for the Management and Control of an Invasive Weed in Europe.

[7] Global Biodiversity Information Facility (GBIF). https://www.gbif.org/occurrence/map?taxon_key=3642949&occurrence_status=present.

[8] Sužiedelytė Visockienė J., Tumelienė E., Malienė V. (2020). Identification of *Heracleum sosnowskyi*-invaded land using earth remote sensing data. Sustainability.

[9] Gubar L., Koniakin S. (2021). Populations of *Heracleum sosnowskyi* and *H. mantegazzianum* (Apiaceae) in Kyiv (Ukraine). Folia Oecolog..

[10] Goncharenko I., Koniakin S., Leshcheniuk O. (2024). Giant hogweeds (*Heracleum mantegazzianum* and *H. sosnowskyi*) in Ukraine: Distribution, ecological and coenotical features. Folia Oecolog..

[11] Baležentienė L., Bartkevičius E. *Heracleum sosnowskyi* (Apiaceae) spread and phytotoxicity. Proceedings of the Sixth International Scientific Conference, Rural Development 2013: Innovations and Sustainability.

[12] Baležentienė L., Bartkevičius E. (2013). Invasion of *Heracleum sosnowskyi* (Apiaceae) at habitat scale in Lithuania. J. Food Agric. Environ..

[13] Baležentienė L., Stankevičienė A., Snieškienė V. (2014). *Heracleum sosnovskyi* (Apiaceae) seed productivity and establishment in different habitats of central Lithuania. Ekologija.

[14] Moravcová L., Gudžinskas Z., Pyšek P., Pergl J., Perglová I., Pyšek P., Cock M.J.W., Nentwig W., Ravn H.P. (2007). Seed Ecology of *Heracleum mantegazzianum* and *H. sosnowskyi*, Two Invasive Species with Different Distributions in Europe. Ecology and Management of Giant Hogweed (Heracleum mantegazzianum).

[15] Grzędzicka E. (2022). Impact of invasive weeds on the diversity and dissimilarity of bird communities in forested areas. Diversity.

[16] Grzędzicka E. (2022). Invasion of the Giant Hogweed and the Sosnowsky’s Hogweed as a Multidisciplinary Problem with Unknown Future—A Review. Earth.

[17] Kalisz S., Kivlin S.N., Bialic-Murphy L. (2021). Allelopathy is pervasive in invasive plants. Biol. Invasions.

[18] Renčo M., Kornobis F., Domaradzki K., Jakubska-Busse A., Jurová J., Homolová Z. (2019). How does an invasive *Heracleum sosnowskyi* affect soil nematode communities in natural conditions?. Nematology.

[19] Renčo M., Baležentienė L. (2015). An analysis of soil-free-living and plant-parasitic nematode communities in three habitats invaded by *Heracleum sosnowskyi* in central Lithuania. Biol. Invasions.

[20] Macías F.A., Durán A.G., Molinillo J.M.G. (2020). Allelopathy: The chemical language of plants. Prog. Chem. Org. Nat. Prod..

[21] Čerevková A., Sarabeev V., Renčo M. (2024). Dataset on soil nematode abundance and composition from invaded and non-invaded grassland and forest ecosystems in Europe. Data Brief.

[22] Shakhmatov E.G., Atukmaev K.V., Makarova E.N. (2016). Structural characteristics of pectic polysaccharides and arabinogalactan proteins from *Heracleum sosnowskyi* Manden. Carbohydr. Polym..

[23] Makarova E.N., Shakhmatov E.G., Belyy V.A. (2016). Structural characteristics of oxalate-soluble polysaccharides of Sosnowsky’s hogweed (*Heracleum sosnowskyi* Manden). Carbohydr. Polym..

[24] Vickackaite V., Pilaityte K., Poskus V. (2025). Extraction, isolation, and purification of furanocoumarins from invasive *Heracleum sosnowskyi*. Separations.

[25] Synowiec A., Kalemba D. (2015). Composition and herbicidal effect of *Heracleum sosnowskyi* essential oil. Open Life Sci..

[26] Politowicz J., Gębarowska E., Proćków J., Pietr S., Szumny A. (2017). Antimicrobial activity of essential oil and furanocoumarin fraction of three *Heracleum* species. Acta Pol. Pharm..

[27] Gordina E.N., Kuznetsov S.P., Golovchenko V.V., Zlobin A.A. (2019). Preliminary structural characteristic of polysaccharides extracted from the callus tissue of Sosnowskyi’s Hogweed (*Heracleum Sosnowskyi* Manden) stem by aqueous ammonium oxalate. Russ. J. Bioorg. Chem..

[28] Gordina E.N., Zlobin A.A., Martinson E.A., Litvinets S.G. (2019). Pectic polysaccharides of callus tissue of the stem of *Heracleum sosnowskyi* Manden. Theor. Appl. Ecol..

[29] Jakubska-Busse A., Śliwiński M., Kobyłka M. (2013). Identification of bioactive components of essential oils in *Heracleum sosnowskyi* and *Heracleum mantegazzianum* (Apiaceae). Arch. Biol. Sci..

[30] Imanly H.A., Serkerov S.V. (2016). Investigation of component composition of roots and fruits *Heracleum sosnowskyi* Manden. Azerbaijan Pharm. Pharmacother. J..

[31] Mishyna M., Laman N., Prokhorov V., Fujii Y. (2015). Angelicin as the principal allelochemical in *Heracleum sosnowskyi* fruit. Nat. Prod. Commun..

[32] Weryszko-Chmielewska E., Chwil M. (2017). Localisation of furanocoumarins in the tissues and on the surface of shoots of *Heracleum sosnowskyi*. Botany.

[33] Del Rio J.A., Diaz L., Garcia-Bernal D., Blanquer M., Ortuno A., Correal E., Moraleda J.M. (2014). Chapter 5—Furanocumarins: Biomolecules of Therapeutic Interest.

[34] Rysiak A., Dresler S., Hanaka A., Hawrylak-Nowak B., Strzemski M., Kováčik J., Sowa I., Latalski M., Wójciak M. (2021). High temperature alters secondary metabolites and photosynthetic efficiency in *Heracleum sosnowskyi*. Int. J. Mol. Sci..

[35] Mahendra C.K., Tan L.T.H., Lee W.L., Yap W.H., Pusparajah P., Low L.E., Tang S.Y., Chan K.G., Lee L.H., Goh B.H. (2020). Angelicin—A furocoumarin compound with vast biological potential. Front. Pharmacol..

[36] Bronikowska J., Szliszka E., Jaworska D., Czuba Z.P., Krol W. (2012). The coumarin psoralidin enhances anticancer effect of tumor necrosis factor-related apoptosis-inducing ligand (TRAIL). Molecules.

[37] Kulikov O.A., Ageev V.P., Brodovskaya E.P., Shlyapkina V.I., Petrov P.S., Zharkov M.N., Yakobson D.E., Maev I.V., Sukhorukov G.B., Pyataev N.A. (2022). Evaluation of photocytotoxicity liposomal form of furanocoumarins Sosnowsky’s hogweed. Chem.-Biol. Interact..

[38] Rassabina A.E., Fedorov M.V. (2025). Analysis of the toxicological profile of *Heracleum sosnowskyi* Manden. metabolites using in silico methods. Plants.

[39] Council of the European Union List of Invasive Alien Species of Union Concern. http://ec.europa.eu/environment/nature/pdf/IAS_brochure_species.pdf.

[40] Gudžinskas Z., Petrulaitis L., Uogintas D., Vaitonis G., Balčiauskas L., Rakauskas V., Arbačiauskas K., Butkus R., Karalius S., Janulaitienė L., Gudžinskas Z., Rašomavičius V. (2023). Invasive and Alien Species in Lithuania.

[41] Stojanović V., Petrović S., Kovačević J., Stojanović D., Bjedo I. (2017). *Heracleum sosnowskyi* Manden. (Apiaceae): A new invasive species in the flora of Serbia. Glas. Šumar. fak..

[42] Koryznienė D., Jurkonienė S., Žalnierius T., Gavelienė V., Jankovska-Bortkevič E., Bareikienė N., Būda V. (2019). *Heracleum sosnowskyi* seed development under the effect of exogenous application of GA_3_. PeerJ.

[43] Žalnierius T., Šveikauskas V., Aphalo P.J., Gavelienė V., Būda V., Jurkonienė S. (2022). Gibberellic Acid (GA_3_) applied to flowering *Heracleum sosnowskyi* decreases seed viability even if seed development is not inhibited. Plants.

[44] Žalnierius T., Laibakojis D., Rapalytė S., Būdienė J., Jurkonienė S. (2025). *HsGA20ox1*, *HsGA3ox1*, and *HsGA2ox1* are involved in endogenous gibberellin regulation within *Heracleum sosnowskyi* ovaries after gibberellin A_3_ treatment. Int. J. Mol. Sci..

[45] Słowiński K., Grygierzec B., Wajs-Bonikowska A., Baran A., Tabor S., Waligórski P., Rys M., Bocianowski J., Synowiec A. (2024). Biochemistry of microwave controlled *Heracleum sosnowskyi* (Manden.) roots with an ecotoxicological aspect. Sci. Rep..

[46] Purmalis O., Klavins L., Niedrite E., Mezulis M., Klavins M. (2025). Invasive plants as a source of polyphenols with high radical scavenging activity. Plants.

[47] Hpoo M.K., Mishyna M., Prokhorov V., Arie T., Takano A., Oikawa Y., Fujii Y. (2020). Potential of octanol and octanal from *Heracleum sosnowskyi* fruits for the control of *Fusarium oxysporum* f. sp. *lycopersici*. Sustainability.

[48] Osipova E.S., Gladkov E.A. (2024). *Heracleum Sosnowskyi* Manden. as a source of valuable chemicals (elimination with utility). Chem. Methodol..

[49] Paramonova K., Chaloupková V., Ivanova T.A. (2024). Invasive *Heracleum sosnowskyi* as a potential feedstock for biorefineries: A review. Ind. Crops Prod..

[50] Baležentienė L. (2012). Inhibitory effects of invasive *Heracleum sosnowskyi* on rapeseed and ryegrass germination. Allelopathy J..

[51] Baležentienė L. (2015). Immediate allelopathic effect of two invasive *Heracleum* species on acceptor-germination. Acta Biol. Univ. Daugavp..

[52] Mishyna M., Pham V.T.T., Fujii Y. (2017). Evaluation of allelopathic activity of *Heracleum sosnowskyi* Manden fruits. Allelopath. J..

[53] Borska E., Kviesis J., Ramata-Stunda A., Nikolajeva V., Ansone-Bertina L., Boroduskis M., Klavins M. (2025). Bioactive lipids and allelopathic potential of the invasive plant *Heracleum sosnowskyi*: Insights into its fatty acid composition, antimicrobial and cytotoxic effects. Front. Pharmacol..

[54] Betekhtina A.A., Ronzhina D.A., Ivanova L.A., Malygin M.V., Ivanov L.A. (2019). Relative growth rate and its components in invasive species *Heracleum sosnowskyi* and congeneric native species *H. sibiricum*. Russ. J. Biol. Invasions.

[55] Malinauskaitė R. (2020). Common oat grayn germination in aqueous extracts of *Heracleum sosnowskyi* leaves. Optim. Ornament. Garden Plant Assort. Technol. Environ..

[56] Bahadori M.B., Dinparast L., Zengin G. (2016). The genus *Heracleum*: A comprehensive review on its phytochemistry, pharmacology, and ethnobotanical values as a useful herb. Compr. Rev. Food Sci. Food Saf..

[57] Kose T.I., Yardimci G.B., Kirci D., Polat D.C., Demirci B., Eryilmaz M., Kilic C.S. (2025). Bioactivities and chemotaxonomy of four *Heracleum* species: A comparative study across plant parts. Pharmaceuticals.

[58] Segneanu A.E., Vlase G., Vlase T., Bejenaru L.E., Mogoşanu G.D., Buema G., Herea D.D., Ciocîlteu M.V., Bejenaru C. (2024). Insight into Romanian wild-grown *Heracleum sphondylium*: Development of a new phytocarrier based on silver nanoparticles with antioxidant, antimicrobial and cytotoxicity potential. Antibiotics.

[59] Inoue A., Taniguchi M., Shibano M., Wang N.-H., Baba K. (2010). Chemical studies on the root of *Heracleum candicans* Wall. (Part 3). J. Nat. Med..

[60] Nakamori T., Taniguchi M., Shibano M., Wang N.-H., Baba K. (2008). Chemical studies on the root of *Heracleum candicans* Wall. J. Nat. Med..

[61] Gao Y., Liu Y., Wang Z.-G., Zhang H.-L. (2014). Chemical constituents of *Heracleum dissectum* and their cytotoxic activity. Phytochem. Lett..

[62] Dincel D., Hatipŏglu S.D., Gören A.C., Topçu G. (2013). Anticholinesterase furocoumarins from *Heracleum platytaenium*, a species endemic to the Ida Mountains. Turk. J. Chem..

[63] Glowniak K., Mroczek T., Zabza A., Cierpicki T. (2000). Isolation and structure elucidation of 5, 7–disubstituted simple coumarins in the fruits of *Heracleum mantegazzianum*. Pharm. Biol..

[64] Taniguchi M., Yokota O., Shibano M., Wang N.-H., Baba K. (2005). Four coumarins from *Heracleum yunngningense*. Chem. Pharm. Bull..

[65] Ageev V.P., Shlyapkina V.I., Kulikov O.A., Zaborovskiy A.V., Tararina L.A. (2022). Qualitative and quantitative analysis of the main psoralen derivatives in the juice of Sosnowsky’s hogweed. Farmaciya (Pharmacy).

[66] Wille W., Thiele J., Walker E.A., Kollmann J. (2013). Limited evidence for allelopathic effects of giant hogweed on germination of native herbs. Seed Sci. Res..

[67] Shtratnikova V.Y., Bogdanov V.P., Schelkunov M.I., Klepikova A.V., Kulbachnaya M.A., Obukhova E.N., Ptitsyna E.V., Ezhova M.A., Penin A.A., Logacheva M.D. (2025). Furanocoumarins in two European species of *Heracleum*: Transcriptomic and metabolomic study. BMC Plant Biol..

[68] Gurina N.S., Lukashov R.I., Kotovich A.V. (2023). Pharmacological properties and component composition of *Heracleum sosnowski* Manden. Med. J..

