# Supplementary material for: Phytochemistry and Allelopathic Properties of Invasive Heracleum sosnowskyi Aqueous Extracts Against Lettuce (Lactuca sativa L.), Perennial Ryegrass (Lolium perenne L.), Timothy (Phleum pratense L.) and White Clover (Trifolium repens L.)
Source: Plants (Basel). 2026 Jan 23;15(3):346. doi: 10.3390/plants15030346 (PMC12899090; doi:10.3390/plants15030346)
Supplement: Supplementary file 1 [file plants-15-00346-s001.zip › plants-4081911-supplementary.pdf]

Phytochemistry and Allelopathic Properties of Invasive *Heracleum sosnowskyi* Aqueous Extracts Against Lettuce (*Lactuca sativa* L.), Perennial Ryegrass (*Lolium perenne* L.), Timothy (*Phleum pratense* L.) and White Clover (*Trifolium repens* L.)

Asta Judžentienė, Aistė Kundrotaitė, Tatjana Charkova and Irena Nedveckytė

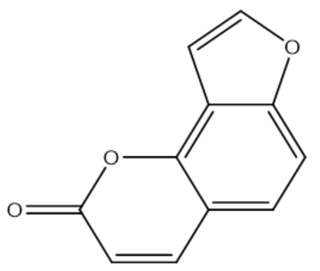

(**1**)

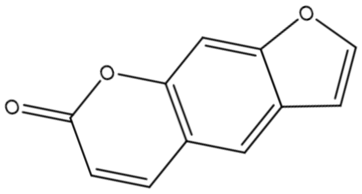

(**2**)

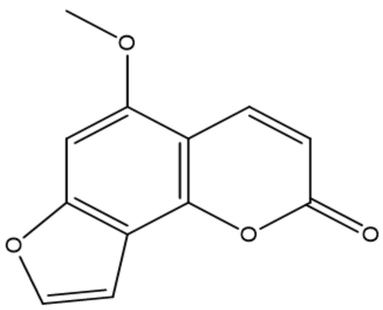

(**3**)

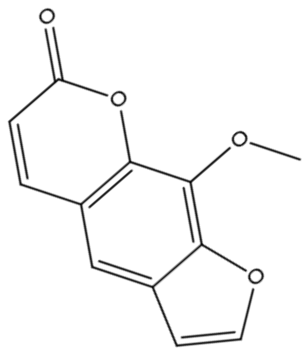

(**4**)

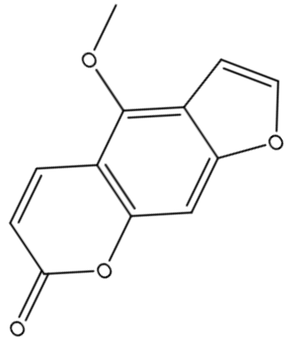

(**5**)

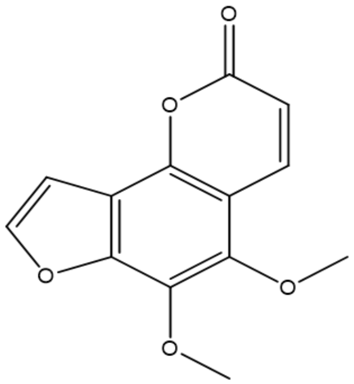

(**6**)

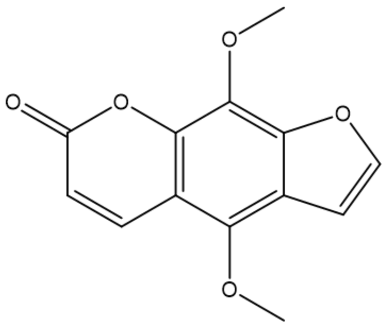

(**7**)

Figure S1. Chemical structures of furan derivatives: 1 – isopsoralen; 2 – ficusin; 3 – isobergapten; 4 – methoxsalen; 5 – bergapten; 6 – pimpinellin and 7 – isopimpinellin, identified in *H. sosnowskyi* extracts were drawn using the ChemDraw (version 16.0) molecule editor. Numbering of compounds (1 – 7) is identical with the numbers indicated in Table 1.

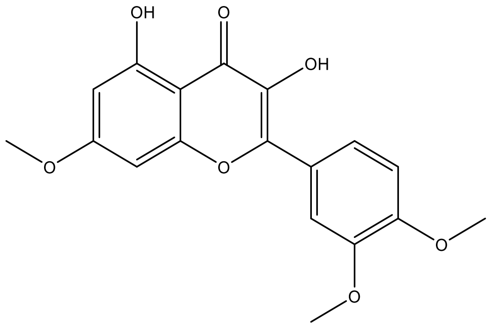

(**22**)

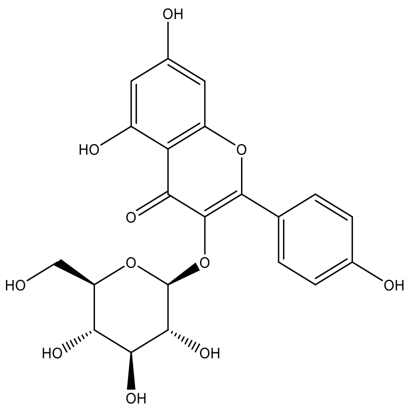

(**23**)

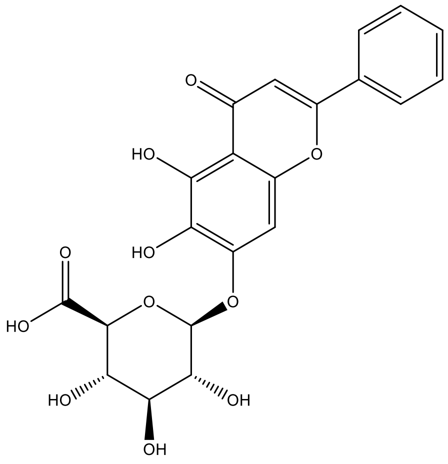

(**24**)

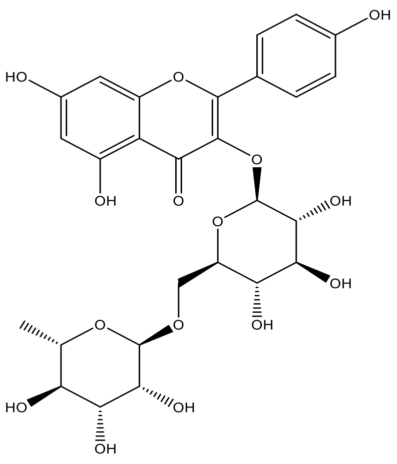

(**25**)

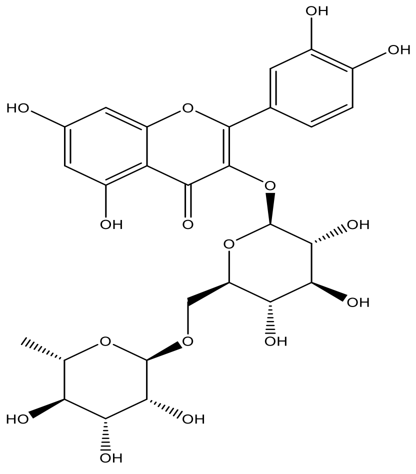

(**26**)

* Numbering of compounds (22– 26) is identical with the numbers indicated in Table 2.

Figure S2. Chemical structures of flavonoids: 22 – quercetin 7,3,4-trimethyl ether; 23 – astragalin; 24 – baicalin; 25 – kaempferol-3-*O*-rutinoside and 26 – rutin, identified in *H. sosnowskyi* extracts.

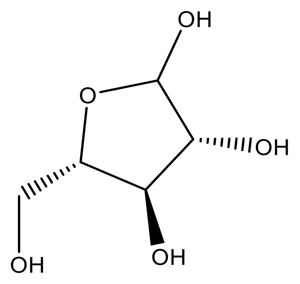

(**37**)

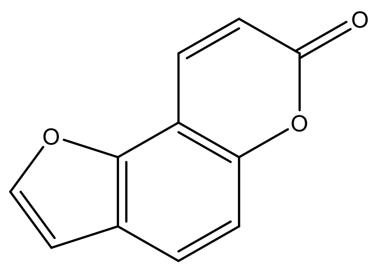

(**38**)

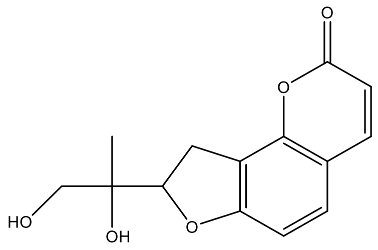

(**39**)

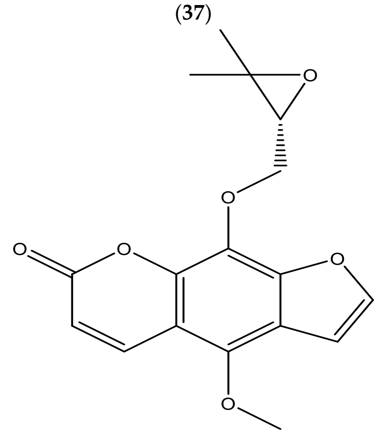

(**40**)

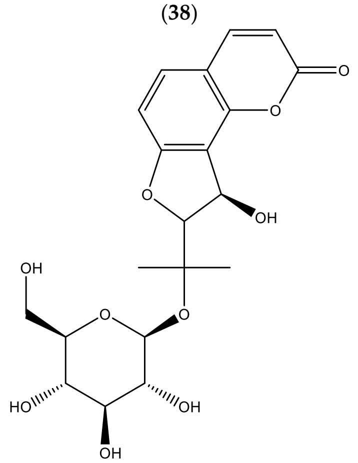

(**41**)

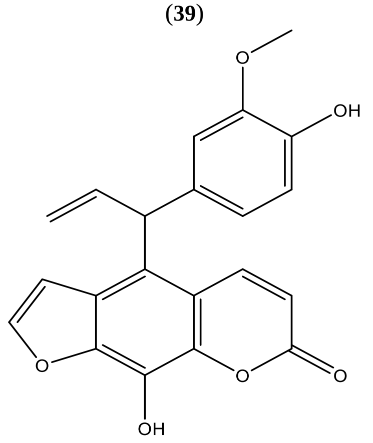

(**42**)

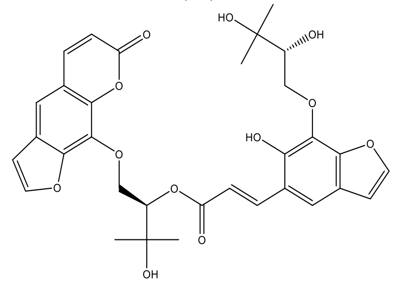

(**43**)

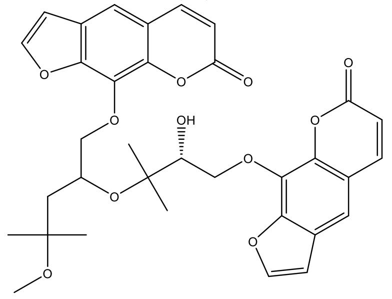

(**44**)

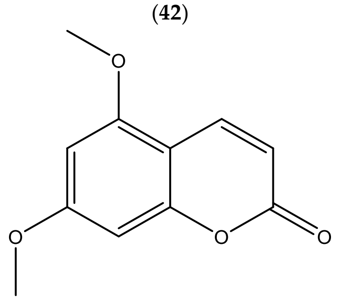

(**45**)

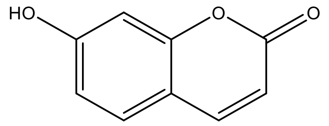

(**46**)

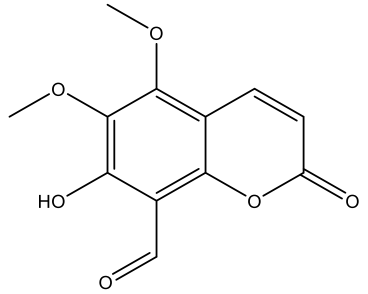

(**47**)

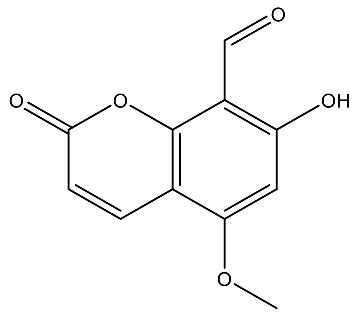

(**48**)

* Numbering of compounds (37 – 48) is identical with the numbers indicated in Table 2.

Figure S3. Chemical structures of furano derivatives (37 – L-arabinofuranose; 38 – bakuchicin; 39 – hermandiol; 40 – byakangelicol; 41 – apterin; 42 – candinol A, 43 – candinol C and 44 – candibirin F) and coumarins (45 – limettin; 46 – umbelliferone; 47 – yunngnin A and 48 – yunngnin B), identified in *H. sosnowskyi* extracts.

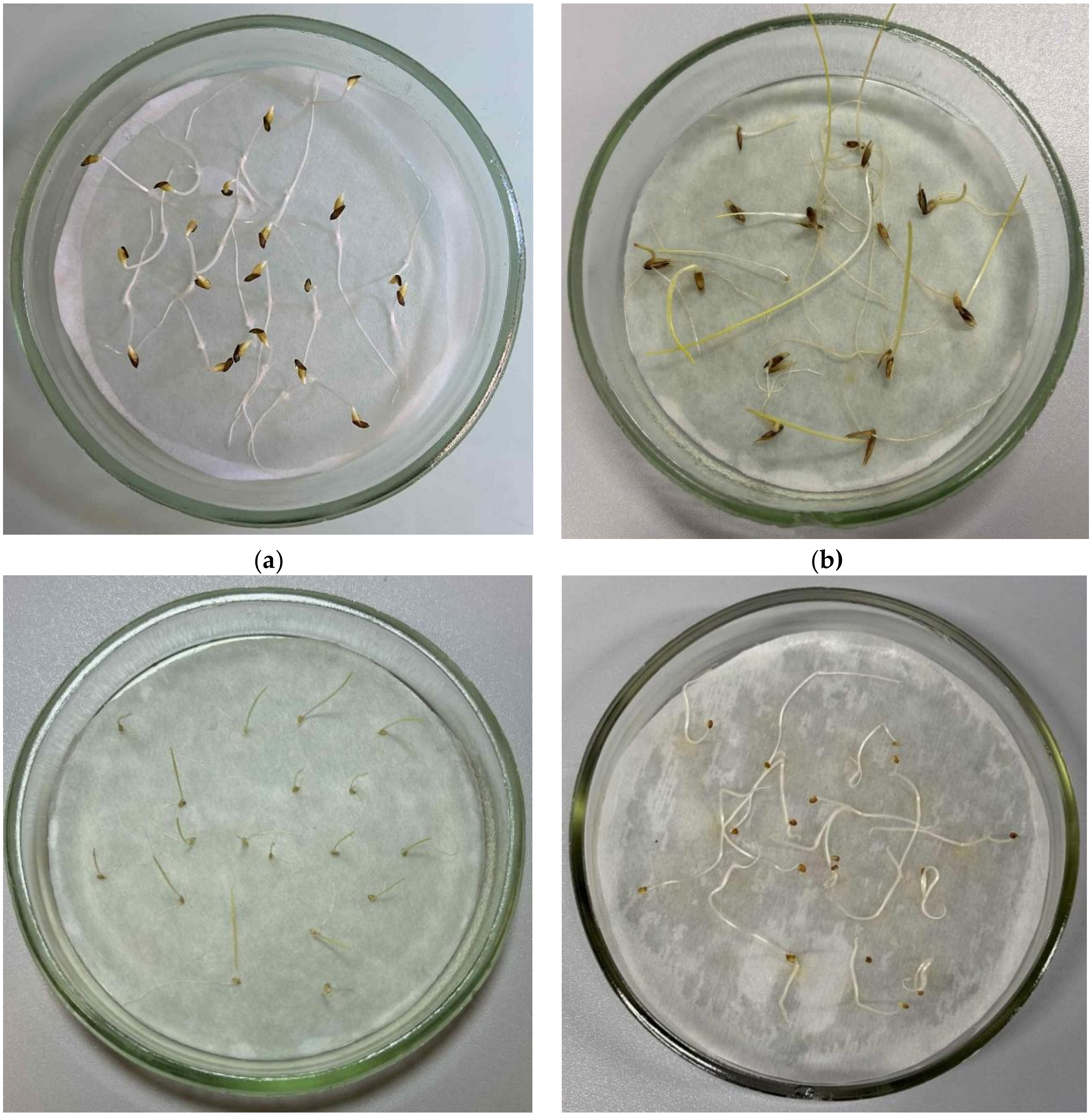

(**a**) (**b**)

(**c**) (**d**)

Figure S4. Photos of control plates of the tested plants seedlings: a – lettuce *(Lactuca sativa* L.), b – perennial ryegrass (*Lolium perenne* L.), c – timothy (*Phleum pratense* L.) and d – white clover (*Trifolium repens* L.).

Table S1. Inhibitory effects of aqueous extracts of *H. sosnowskyi* inflorescences, leaves and roots on the germination rate (GR, %) of *L. sativa, L. perenne, P. pratense* and *T. repens* seeds. Data are presented as the means of nine treatments (n = 180) ± SD (Standard Deviation). Different letters (a, b, c and d) with the germination rate values indicate statistically significant differences between groups ($p < 0.05$), determined by the Kruskal–Wallis test followed by the Mann-Whitney pairwise test.

Germination rate (%) of *L. sativa* seeds				
Control 94.44 ± 5.98 (a)	Relative concentration	Flower extract	Leaf extract	Root extract
	0.1	87.22 ± 6.43 (a)	89.44 ± 8.31 (a)	88.33 ± 7.45 (ab)
	0.5	57.78 ± 17.12 (b)	55.00 ± 12.29 (b)	80.56 ± 4.37 (b)
	1.0	32.78 ± 3.43 (b)	25.56 ± 10.60 (c)	36.67 ± 10.61 (c)
**Germination rate (%) of *L. perenne* seeds**				
Control 87.78 ± 3.42 (a)	Relative concentration	Flower extract	Leaf extract	Root extract
	0.1	63.33 ± 2.36 (b)	66.67 ± 7.58 (b)	71.11 ± 6.29 (b)
	0.5	16.67 ± 5.93 (c)	31. 67 ± 9.43 (c)	52.78 ± 5.13 (c)
	1.0	6.11 ± 0.79 (c)	15.56 ± 3.16 (d)	41.11 ± 7.06 (c)
**Germination rate (%) of *P. pratense* seeds**				
Control 92.78 ± 4.16 (a)	Relative concentration	Flower extract	Leaf extract	Root extract
	0.1	90.00 ± 4.71 (a)	78.89 ± 3.14 (b)	82.78 ± 6.43 (a)
	0.5	22.78 ± 2.83 (b)	37.78 ± 5.50 (c)	28.33 ± 5.93 (b)
	1.0	0.00	22.22 ± 0.79 (d)	4.44 ± 1.57 (c)
**Germination rate (%) of *T. repens* seeds**				
Control 96.11 ± 5.15 (a)	Relative concentration	Flower extract	Leaf extract	Root extract
	0.1	70.56 ± 11.41 (b)	46.11 ± 14.33 (b)	59.44 ± 7.97 (b)
	0.5	8.33 ± 1.18 (c)	0.56 ± 0.21 (c)	30.00 ± 1.36 (c)
	1.0	2.78 ± 1.57 (d)	0.00	24.44 ± 8.85 (c)

Table S2. Seed vigor index (VI) of lettuce *(Lactuca sativa* L.), perennial ryegrass (*Lolium perenne* L.), timothy (*Phleum pratense* L.) and white clover (*Trifolium repens* L.).

**Aqueous extracts of *H. sosnowkyi* flowers**				
**Treatments**	**Seed Vigor index (VI)**			
	**Lettuce**	**Ryegrass**	**Timothy**	**Clover**
**Control**	3433.84	2945.90	1822.20	3961.65
**0.1**	1794.99	704.86	375.30	1080.97
**0.5**	290.63	71.85	20.27	10.33
**1.0**	71.46	12.89	0.00	2.17
**Aqueous extracts of *H. sosnowkyi* leaves**				
**Treatments**	**Seed Vigor index (VI)**			
	**Lettuce**	**Ryegrass**	**Timothy**	**Clover**
**Control**	3433.84	2945.90	1822.20	3961.65
**0.1**	1932.80	1582.08	620.08	709.17
**0.5**	165.55	98.18	73.29	0.12
**1.0**	43.20	20.07	42.22	0.00
**Aqueous extracts of *H. sosnowkyi* roots**				
**Treatments**	**Seed Vigor index (VI)**			
	**Lettuce**	**Ryegrass**	**Timothy**	**Clover**
**Control**	3433.84	2945.90	1822.20	3961.65
**0.1**	2598.67	1836.06	1007.43	953.42
**0.5**	861.99	586.39	69.69	71.40
**1.0**	118.08	256.94	7.06	50.59

Table S3. Calculation of germination rate (GR) and vigor index (VI).

**Index**	**Equation**
Germination rate (GR), %	GR = final number of germinated seeds after 5 days of incubation/20 × 100%
Vigor index (VI)	VI = PL (mm) × GR (%)<br>PL- Plant length
